# *In Vivo* Gene Editing of Muscle Stem Cells with Adeno-Associated Viral Vectors in a Mouse Model of Duchenne Muscular Dystrophy

**DOI:** 10.1016/j.omtm.2020.09.016

**Published:** 2020-09-28

**Authors:** Jennifer B. Kwon, Adarsh R. Ettyreddy, Ashish Vankara, Joel D. Bohning, Garth Devlin, Stephen D. Hauschka, Aravind Asokan, Charles A. Gersbach

**Affiliations:** 1 University Program in Genetics and Genomics, Duke University Medical Center, Durham, NC 27710, USA; 2 Center for Advanced Genomic Technologies, Duke University, Durham, NC 27708, USA; 3 Department of Biomedical Engineering, Duke University, Durham, NC 27708, USA; 4 Department of Surgery, Duke University Medical Center, Durham, NC 27710, USA; 5 Department of Biochemistry, University of Washington, Seattle, WA, USA; 6 Regeneration Next Initiative, Duke University Medical Center, Durham, NC 27710, USA

**Keywords:** dmd, satellite, pax7, gene-editing, crispr, aav, muscle, promoters

## Abstract

Delivery of therapeutic transgenes with adeno-associated viral (AAV) vectors for treatment of myopathies has yielded encouraging results in animal models and early clinical studies. Although certain AAV serotypes efficiently target muscle fibers, transduction of the muscle stem cells, also known as satellite cells, is less studied. Here, we used a Pax7nGFP;Ai9 dual reporter mouse to quantify AAV transduction events in satellite cells. We assessed a panel of AAV serotypes for satellite cell tropism in the *mdx* mouse model of Duchenne muscular dystrophy and observed the highest satellite cell labeling with AAV9 following local or systemic administration. Subsequently, we used AAV9 to interrogate CRISPR/Cas9-mediated gene editing of satellite cells in the Pax7nGFP;mdx mouse. We quantified the level of gene editing using a Tn5 transposon-based method for unbiased sequencing of editing outcomes at the *Dmd* locus. We also found that muscle-specific promoters can drive transgene expression and gene editing in satellite cells. Lastly, to demonstrate the functionality of satellite cells edited at the *Dmd* locus by CRISPR *in vivo*, we performed a transplantation experiment and observed increased dystrophin-positive fibers in the recipient mouse. Collectively, our results confirm that satellite cells are transduced by AAV and can undergo gene editing to restore the dystrophin reading frame in the *mdx* mouse.

## Graphical Abstract

**Figure:**
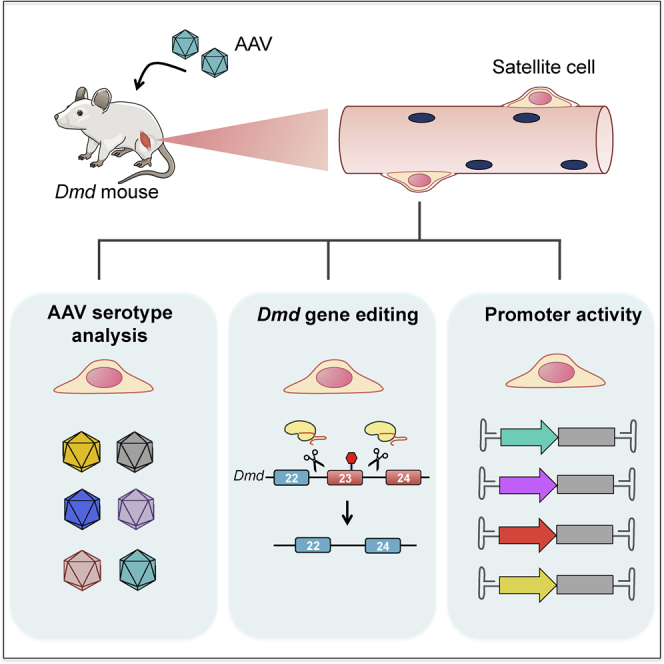

## Teaser

This study supports previous evidence of AAV transduction and CRISPR/Cas9-mediated gene editing of muscle satellite cells following local and systemic delivery. New insights include differences between AAV capsid serotypes and tissue-specific promoters driving Cas9 expression. Additionally, quantification of various gene-editing outcomes in satellite cells is provided using unbiased assays.

## Introduction

Duchenne muscular dystrophy (DMD) is a debilitating genetic disease that affects 1 in 5,000 live male births and is characterized by the lack of functional dystrophin protein, resulting in progressive lethal skeletal muscle degeneration.^1^ Skeletal muscle degeneration stimulates the satellite stem cell population to proliferate and give rise to new myofibers. In DMD, satellite cells are overwhelmed by the constant demand for muscle regeneration. Excessive proliferation results in replicative senescence, and the satellite cell regenerative capacity gradually declines, giving way to relentless muscle degeneration accompanied by fibrosis and adipose deposition.^2^ Although clinical advancements have been made for treatment of this disease, a cure remains to be developed.^3^ Due to its genetic nature, DMD is an excellent candidate for therapeutic gene editing,^4^ and several groups have recently demonstrated successful CRISPR/Cas9-based correction of the dystrophin gene in animal models.^5^, ^6^, ^7^, ^8^, ^9^, ^10^, ^11^ To deliver CRISPR/Cas9 to the muscle, gene-editing constructs are most commonly packaged in adeno-associated viruses (AAVs), which are effective gene-delivery vectors used in over 100 clinical trials with three approved therapies in the United States or Europe.^12^ Because satellite cells continuously replenish skeletal muscle in response to tissue damage, the genetic correction of a population of these self-renewing cells could generate a sustained source of therapeutic gene production. In fact, because episomal AAV vectors are lost by dilution following cell division,^13^ permanent correction of the genomic copy of mutated genes in satellite cells is a particularly compelling advantage of gene-editing technologies. Furthermore, efficient targeting of satellite cells with AAV vectors *in vivo* would enable many studies of the function and regulation of satellite cell biology within the native environment.

An early study investigating AAV transduction of satellite cells based on immunohistochemical staining and delivery of GFP found little or no vector transduction despite high transduction in skeletal myocytes.^14^ By using dual reporter mice, in which satellite cells are permanently marked by Cre-mediated recombination following transduction of AAV even after the AAV vector is lost, more recent studies have demonstrated that AAV transduction in satellite cells does occur at significant frequencies.^6^^,^^15^^,^^16^ Furthermore, they show that detectable levels of gene editing occur in satellite cells at the *Dmd* locus following AAV9-cytomegalovirus (CMV)-CRISPR treatment.^6^^,^^16^ Here, we expanded upon these studies by profiling AAV serotypes for satellite cell targeting, quantifying the level of gene editing, demonstrating *in vivo* functionality of gene-edited cells, and measuring the activity of muscle-specific promoters in satellite cells.

With the use of a Pax7nGFP;Ai9 dual reporter mouse, we also found significant AAV transduction of satellite cells in both *mdx* and wild-type (WT) mice. We tested multiple AAV serotypes with intramuscular and systemic injections and found AAV8 and AAV9 have the highest tropism to satellite cells. We then treated Pax7nGFP;mdx mice with a dual-AAV9 CRISPR system designed to excise exon 23 in the *mdx* mouse. To quantify gene-editing levels in satellite cells without introducing *ex vivo* culturing artifacts, we isolated satellite cells 8 weeks after treatment with AAV9-CMV-CRISPR constructs and immediately harvested genomic DNA for analysis. We used an unbiased sequencing approach to quantify gene-editing outcomes, including exon deletions, indels, inversions, and integration of AAV inverted terminal repeats (ITRs). To investigate continued therapeutic efficacy from satellite cell-genome editing, we implemented a serial injury model to induce muscle degeneration with concomitant loss of therapeutic vectors and found that mice treated with CRISPR retained dystrophin expression after three rounds of injury. We also directly demonstrate that CRISPR-corrected satellite cells can give rise to new dystrophin fibers in a transplantation assay. Finally, we demonstrate activity and verify gene editing with muscle-specific promoters driving *Staphylococcus aureus* Cas9 (SaCas9) expression in satellite cells. Collectively, these results confirm that satellite cells are transduced by AAV and undergo gene editing by CRISPR, which could facilitate enduring therapeutic effects for DMD.

## Results

### Profiling AAV Serotypes for Targeting Efficiency of Satellite Cells

**Figure 1:**
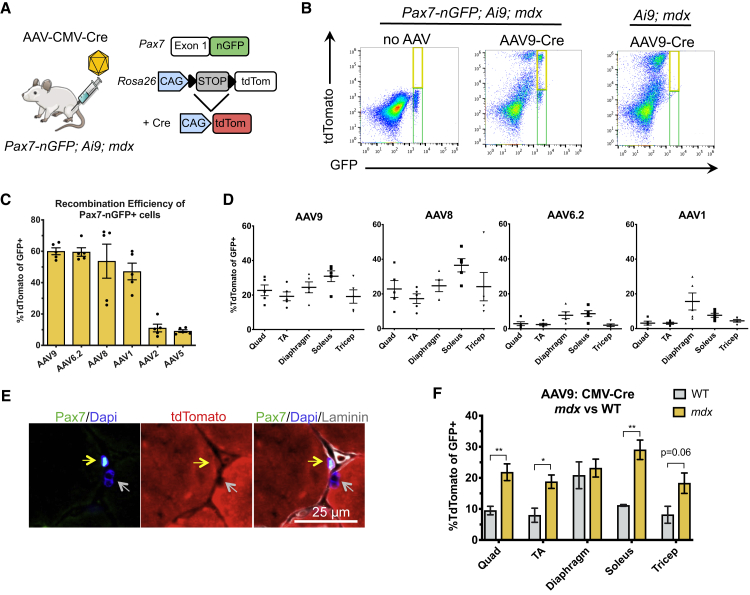
A Dual Reporter Mouse to Quantify AAV Transduction in Satellite Cells (A) Schematic illustration of the dual reporter mouse harboring a knock-in nuclear GFP (nGFP) at the *Pax7* locus and CAG-LSL-tdTomato at the *Rosa26* locus. Cre-mediated recombination results in tdTomato expression. (B) FACS plots and controls used for establishing the gating strategy. The green gate identifies Pax7-nGFP^+^ cells, whereas the yellow gate identifies Pax7nGFP^+^/tdTomato^+^ cells. (C) Recombination efficiency of Pax7-nGFP^+^ cells after local injections of Cre packaged in a panel of AAV serotypes (mean ± SEM, n = 5 mice). (D) Recombination efficiency in Pax7nGFP^+^ cells from various skeletal muscle groups after systemic injections of AAV-Cre (mean ± SEM, n = 5 mice). (E) Representative immunofluorescence staining of a Pax7^+^/tdTomato^+^ cell (yellow arrow) contrasted by a Pax7^−^/tdTomato^−^ nucleus (gray arrow). (F) Systemic injection of AAV9-CMV-Cre in *mdx* versus wild-type mice demonstrates higher transduction of satellite cells in a dystrophic muscle context (mean ± SEM, n = 5 mice).

The Ai9 mouse allele harbors a CAG-loxP-STOP-loxP-tdTomato expression cassette at the *Rosa26* locus.^17^ Excision of the stop cassette by the Cre recombinase leads to permanent labeling of target cells with expression of the tdTomato fluorescent protein. We crossed the Ai9 mice to the Pax7nGFP mice, in which a nuclear-localized GFP (nGFP) is knocked into the first exon of *Pax7* to specifically label satellite cells.^18^ By delivering the Cre recombinase via an AAV vector, tdTomato expression labels cells transduced by the AAV (Figure 1A). Therefore, any GFP^+^ cells that coexpress tdTomato represent satellite cells that were transduced with the AAV vector (Figure 1B). We injected AAV1, AAV2, AAV5, AAV6.2 (AAV6 with a point mutation increasing transduction efficiency^19^), AAV8, and AAV9 serotypes encoding CMV-Cre into the tibialis anterior (TA) muscles of Pax7nGFP;Ai9;mdx mice at equivalent doses of 4.72E+11 vector genome (vg). Muscles were harvested 8 weeks after injection, and the tissue was dissociated to single cells for immediate analysis by flow cytometry. We found that AAV9, AAV6.2, and AAV8 marked the Pax7nGFP^+^ cells most efficiently, leading to tdTomato expression in ~60% of nGFP^+^ cells (Figure 1C). We then assessed the top four performing AAV serotypes by systemic AAV-CMV-Cre administration via tail-vein injection at equivalent doses of 2E+12 vg. We harvested various skeletal muscle types and found that following systemic injection, AAV8 and AAV9 clearly outperformed AAV6.2 and AAV1 with significant targeting of Pax7-GFP^+^ cells, ranging from 20% to 30% for various muscle types (Figure 1D). Correct colocalization of tdTomato and Pax7 *in vivo* was confirmed by immunofluorescence staining of tissue sections (Figure 1E).

Because satellite cells are activated and proliferative in dystrophic muscle relative to normal tissues,^20^ we also injected AAV9-CMV-Cre systemically in Pax7nGFP;Ai9;WT mice to investigate the role of the dystrophic environment on AAV transduction of satellite cells. Interestingly, we found significantly different transduction efficiencies of satellite cells in *mdx* versus WT mice for all muscle tissues tested except the diaphragm (Figure 1F), perhaps related to the activated state of satellite cells in regenerating dystrophic muscles.

### AAV9-CMV-CRISPR Constructs Target Satellite Cells for Gene Editing *In Vivo*

**Figure 2:**
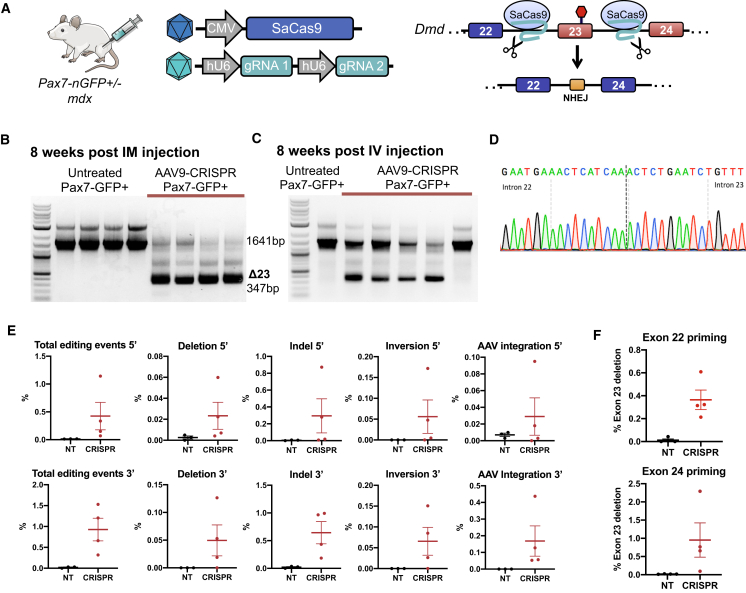
AAV9-CRISPR Induces Gene Editing of Satellite Cells at the *Dmd* Locus in *mdx* Mice (A) Pax7nGFP/mdx mice were injected with AAV9-CRISPR designed to excise exon 23 from the *Dmd* locus to restore the reading frame. (B) PCR across the genomic deletion region in satellite cells isolated from TA muscles shows a smaller 347-bp PCR band corresponding to excision of exon 23 present only in CRISPR-treated cells. (C) Isolated satellite cells from systemically injected mice also demonstrate deletion bands corresponding to excision of exon 23 in four out of five mice. (D) Sanger sequencing of the smaller 347-bp PCR band demonstrates perfect ligation of gRNA target sites in intron 22 and intron 23. (E) Unbiased Tn5 tagmentation-based sequencing of the targeted region around exon 23 of the *Dmd* locus from either the 5′ or 3′ direction in satellite cell genomic DNA after AAV9-CRISPR local administration quantifies the level of editing events for various gene-editing outcomes. (F) Unbiased Tn5 tagmentation-based sequencing of satellite cell mRNA after AAV9-CMV-CRISPR local administration quantifies the level of exon 23 deletion.

Next, we used the Pax7nGFP;mdx mouse to assess the level of gene editing in satellite cells with a dual AAV9-CRISPR strategy consisting of one AAV9 vector encoding CMV-driven SaCas9 and the other AAV9 vector encoding two guide RNAs (gRNAs) designed to excise exon 23 from the *Dmd* gene in *mdx* mice, as previously described^5^ (Figure 2A). The SaCas9 and gRNA AAV vectors were premixed in equivalent viral titers of 1E+12 vg/vector and injected into the TA muscle. Control mice received an injection of an equal volume of PBS to the TA. At 8 weeks after injection, muscle was harvested for enzymatic dissociation and satellite cell sorting. Genomic DNA was isolated from sorted cells, and a nested PCR reaction was performed across exon 23 to visualize the rare and smaller deletion band in satellite cells isolated from CRISPR-treated muscle (Figure 2B). Systemic delivery of AAV9-CMV-CRISPR at 5E+12 vg/vector was also performed, and satellite cells were isolated from hind-limb muscles and the diaphragm 8 weeks after treatment. A deletion band could also be detected from satellite cells after systemic intravenous delivery in the majority of samples (Figure 2C). Sanger sequencing of the gel-extracted deletion band confirms exon 23 deletion (Figure 2D).

To quantify the level of gene editing in satellite cells, we adapted a Tn5 transposon-based DNA tagmentation protocol for unbiased sequencing, as previously described.^21^ With the use of this method, we quantified gene-editing outcomes, including exon deletion, indels at either gRNA target site, inversions, and integration of AAV ITRs in satellite cells, 8 weeks after intramuscular injection of AAV9-CMV-CRISPR (Figure 2E). The various editing outcomes ranged from ~0.01% to 1% in satellite cells, with indels at single gRNA sites being the most common outcome. We also applied this method to cDNA from these cells to quantify the level of exon 23 deletion in dystrophin transcripts, which ranged from ~0.4% to 1% (Figure 2F). These editing frequencies in satellite cells are ~10-fold lower than what we previously reported in the treated bulk muscle tissue^21^ and similarly observed here (Figures S1A and S1B).

### Muscle-Specific Promoters Are Active in Satellite Cells

**Figure 3:**
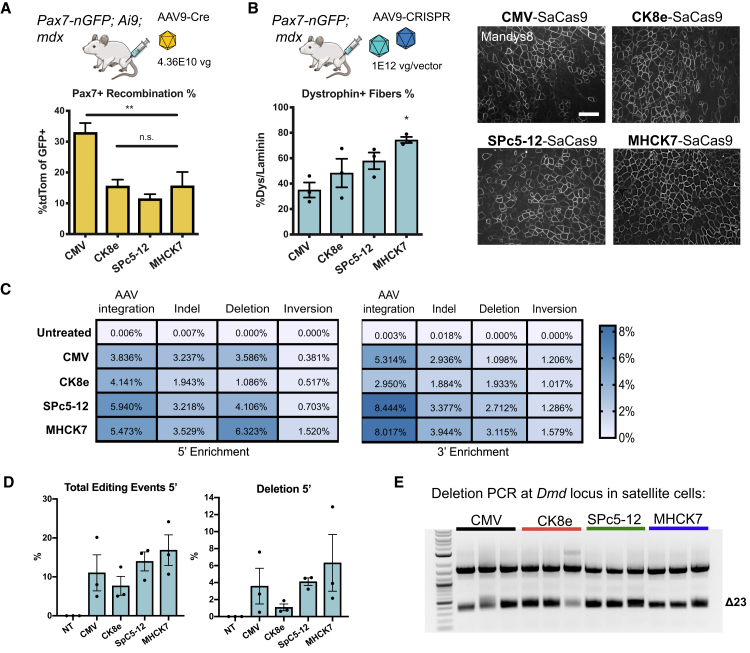
Muscle-Specific Promoters Are Active in Satellite Cells (A) Muscle-specific promoters driving Cre expression were packaged into AAV9 and delivered in equal doses by intramuscular injection into Pax7nGFP;Ai9;mdx mice. Recombination efficiency of satellite cells was highest in CMV-driven Cre (mean ± SEM, n = 3–4 mice). (B) Pax7nGFP/mdx mice were injected with AAV9-gRNAs targeted to intronic sequences 3′ and 5′ of exon 23 along with AAV9 encoding SaCas9 driven by CMV, CK8e, SPc5-12, or MHCK7 promoters. 8 weeks after intramuscular injections, the TA muscle was harvested and dystropin-positive fibers quantified by immunofluorescence staining. Scale bar = 200 μM. (C) Unbiased sequencing of TA muscle genomic DNA after local administration of AAV9-CRISPR harboring various muscle-specific promoters or CMV quantifies the level of editing events for various gene-editing outcomes. (D) Total editing events and deletion events are quantified by unbiased sequencing of bulk muscle after local AAV9-CRISPR treatment with various promoters (mean ± SEM, n = 3 mice). (E) PCR of genomic DNA from sorted satellite cells demonstrates the excision of exon 23 across mice injected with SaCas9 driven by CMV or muscle-specific promoters. p value determined by one-way ANOVA, followed by Tukey’s post hoc test (mean ± SEM, n = 3 mice).

Next, we sought to define the recombination efficiency in satellite cells when Cre is driven by muscle-specific promoters as opposed to a constitutive CMV promoter. Because many commonly used AAV vectors display broad-tissue tropism, clinical trials are moving forward with tissue-specific promoters when available to avoid off-target expression of transgenes. CMV-driven Cas9 expression has been shown to elicit an immune response in adult mice and can cause gene editing in nonmuscle tissue.^21^ The restriction of Cas9 expression to muscles can reduce the risk of off-target genome-editing effects and could minimize the elicitation of an immune response.^22^ Although muscle-specific promoters are designed to target skeletal and heart muscle efficiently, the extent of expression in satellite cells is presumed to be inefficient.^9^ To determine the efficiency of gene expression in satellite cells with our dual reporter system, we delivered 4E+10 vg of AAV9 encoding the ubiquitous CMV promoter or the muscle-specific CK8e,^23^ SPc5-12,^24^ or MHCK7^25^ promoters driving Cre recombinase expression to the TA muscle of Pax7nGFP;Ai9;mdx mice. Compared to CMV (33%), the efficiency of recombination was about one-half for MHCK7 (15.6%) and CK8e (15.6%) and one-third for SpC5-12 (11.5%), suggesting that these muscle-specific promoters are active in satellite cells, albeit to a lesser degree than CMV (Figure 3A).

To compare gene-editing efficiencies in ubiquitous versus muscle-specific promoters, we drove SaCas9 expression with CMV, CK8e, SPc5-12, or MHCK7 promoters and delivered AAV9-CRISPR constructs intramuscularly at equivalent viral doses. We compared dystrophin restoration at the bulk muscle level among the different promoters, and immunofluorescence staining of TA muscle sections revealed higher numbers of dystrophin^+^ fibers in muscles treated with AAV-CRISPR harboring MHCK7 (73%), SPc5-12 (53%), and CK8e (48.3%) promoters compared to CMV (35%) (Figure 3B). With the use of the unbiased Tn5 tagmentation-based method, we also quantified the level of gene-editing outcomes across these different promoters in the bulk muscle (Figures 3C and 3D). We found the highest occurrence of deletions in the MHCK7-SaCas9-treated mice, followed by SPc5-12. To determine if gene editing can be accomplished in satellite cells with muscle-specific promoters, we isolated nGFP^+^ satellite cells from treated mice and performed a nonquantitative nested PCR across the *Dmd* locus. Exon 23 deletion bands were detected in all conditions (Figure 3E).

### Sustained Dystrophin Expression in AAV-CMV-CRISPR-Treated Muscle after Serial Injuries

**Figure 4:**
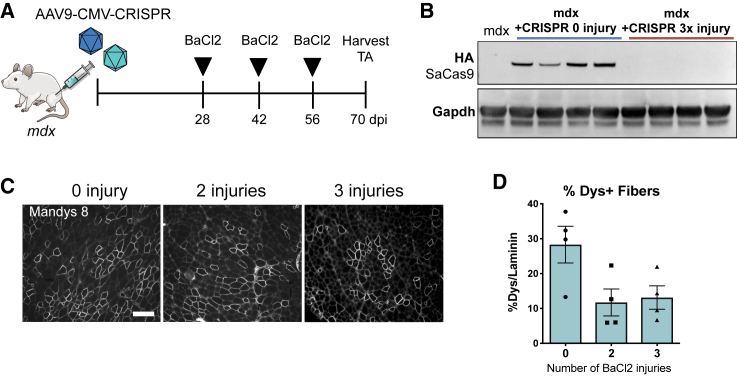
Serial Injury of TA Muscle Treated with AAV9-CMV-CRISPR Demonstrates Sustained Expression of Dystrophin after Loss of AAV Vector Expression (A) Schematic illustration of serial injury strategy. *mdx* mice were treated with AAV9-CMV-CRISPR constructs by intramuscular injection. 4 weeks later, mice were injured with 50 μL of 1.2% BaCl_2_ to induce muscle degeneration and regeneration. BaCl_2_ injections were administered a total of 2 or 3 times with a 2-week recovery period between each injection. (B) Western blot of the HA epitope tag on the C terminus of SaCas9 shows clearance of SaCas9 after three BaCl_2_ injuries. (C) Representative immunofluorescence images of dystrophin restoration in *mdx* mice treated with AAV9-CMV-CRISPR and injured 0, 2, or 3 times with BaCl_2_. Scale bar = 200 μM. (D) Quantification of dystrophin^+^ fibers after AAV9-CMV-CRISPR treatment with 0, 2, or 3 injuries with BaCl_2_ (mean ± SEM, n = 4 mice).

Targeting satellite cells for dystrophin gene correction could provide a self-renewing source of dystrophin-expressing cells that might provide continued therapeutic effects even after loss of the episomal AAV vector. To investigate the long-term contribution of dystrophin-corrected satellite cells, we injected TA muscles of *mdx* mice with AAV9-CRISPR constructs with CMV promoter-driving SaCas9 and monitored dystrophin expression. Because the *mdx* mouse model does not recapitulate the severity of the human DMD-degenerative phenotype, we accelerated muscle degeneration and regeneration by implementing a serial injury strategy. 4 weeks after the initial injection of AAV9-CMV-CRISPR constructs, mice were injected with 50 μL of 1.2% barium chloride (BaCl_2_) to induce muscle injury every 2 weeks for a maximum of 6 weeks (Figure 4A). This dose of BaCl_2_ injury to the TA induces necrosis in over 80% of muscle fibers, 18 h after injury in WT mice.^26^ We thus hypothesized that 2 or 3 rounds of injury would be sufficient to induce degeneration of CRISPR-treated myofibers, followed by regeneration of new myofibers from satellite cells. After 2 or 3 injuries, we could no longer detect SaCas9 protein in the TA muscle by western blot, indicating loss of the AAV9 vectors (Figure 4B). Despite loss of vector, we observed maintenance of dystrophin expression over three rounds of regeneration by immunofluorescence staining (Figure 4C). Quantification of dystrophin^+^ fibers as a fraction of all fibers (laminin^+^) indicates an initial restoration of dystrophin in 28.3% of fibers that decreased to ~10% after either 2 or 3 injuries (Figure 4D). This observed maintenance of dystrophin protein after loss of SaCas9 suggests that edited satellite cells may serve as a long-term reservoir for dystrophin production. It is also possible that dystrophin restoration in satellite cells is conferring selective advantage, given that dystrophin has been reported to play a role in satellite cell maintenance^27^.

### Serial Transplantation of CRISPR-Corrected Satellite Cells Contributes to Regeneration of Dystrophin^+^ Myofibers

**Figure 5:**
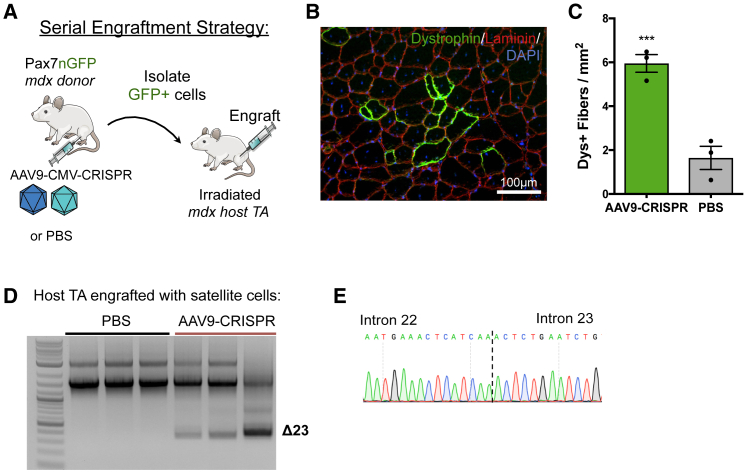
Serial Transplantation of CRISPR-Edited Satellite Cells from *mdx* Mice Contributes to Muscle Regeneration (A) Schematic illustration of the serial engraftment strategy. Pax7nGFP;mdx donor mice were injected with AAV9 vectors encoding CMV-SaCas9 and exon23-targeting gRNAs or PBS. 8 weeks later, GFP^+^ cells were isolated by flow cytometry and immediately injected into *mdx* host mice that had their left hindlimb irradiated 2 days prior. 4 weeks later, the host TA muscles were isolated for analysis. (B) Representative images of immunofluorescence staining for dystrophin in TA muscles from *mdx* host mice injected with satellite cells from mice treated with AAV9-CMV-CRISPR. (C) Quantification of dystrophin^+^ fibers per square millimeter in host TA muscles injected with satellite cells isolated from donor mouse muscles treated with either AAV9-CMV-CRISPR constructs or PBS (mean ± SEM, n = 3 mice). (D) PCR of host genomic DNA extracted from TA muscles shows exon 23 deletion bands are present only in mice injected with satellite cells from AAV9-CMV-CRISPR-treated donors. (E) Sanger sequencing of the deletion band demonstrates perfect ligation of intron 22 to intron 23.

To demonstrate that gene-edited satellite cells can give rise to dystrophin^+^ myofibers, we performed a serial transplantation study (Figure 5A). Pax7nGFP;mdx mice were injected in the hindlimb with either AAV9-CMV-CRISPR constructs or PBS. 8 weeks later, the injected muscles were harvested, and ~30,000 GFP^+^ satellite cells were sorted by fluorescence-activated cell sorting (FACS). Sorted cells were immediately transplanted into the TA muscle of an otherwise untreated *mdx* host mouse, which received 18 Gy irradiation, 2 days prior to incapacitate host satellite cells.^28^ The host mice were immunosuppressed with daily intraperitoneal (i.p.) injections of tacrolimus. At 4 weeks postengraftment, the TA muscles were harvested and analyzed for dystrophin expression. Patches of dystrophin^+^ fibers were observed in the host TA muscles injected with CRISPR-corrected satellite cells (Figure 5B). *mdx* host mice that were injected with satellite cells harvested from PBS-injected Pax7nGFP;mdx donor mice displayed 1.46 ± 0.41 dystrophin^+^ fibers per square millimeter, which is similar to the number of revertant fibers found in *mdx* mice of the same age group.^29^ In contrast, *mdx* mice that were injected with satellite cells harvested from CRISPR-injected *mdx* mice displayed 5.95 ± 0.40 dystrophin^+^ fibers per square millimeter (Figure 5C), suggesting that transplantation of CRISPR-corrected satellite cells led to an increase in dystrophin^+^ fibers. When we performed a deletion PCR across the *Dmd* locus, we saw a deletion band only in genomic DNA from the host TA that was injected with CRISPR-treated satellite cells, indicating that the dystrophin^+^ fibers observed in that group are produced from gene-edited cells (Figure 4D).

## Discussion

In this study, we demonstrate that AAV can efficiently transduce satellite cells *in vivo* using a sensitive Cre/lox-based dual-reporter mouse. We test a series of commonly used AAV serotypes that exhibit unique tissue tropism to conclude that AAV9 and AAV8 are most suitable for satellite cell transduction in both local injections as well as systemic tail-vein injections (Figure 1). These results support the conclusions of recent papers that utilized a similar strategy^6^^,^^15^^,^^16^ and contrast a previous report that satellite cells are not efficiently transduced by AAV.^14^

Interestingly, we found higher AAV9 transduction in *mdx* mice compared to WT mice (Figure 1F). Satellite cells are heterogeneous and can exist in both quiescent and activated states. Due to the constant demand for regeneration, *mdx* mice have a higher number of activated satellite cells^20^^,^^30^ that express a set of surface markers that distinguish them from quiescent satellite cells.^31^ This difference in surface markers may influence their ability to be transduced by AAV. The congruence in diaphragm satellite cell transduction efficiencies in WT versus *mdx* mice may be due to the fact that the diaphragm is functionally and morphologically distinct from limb muscles. The diaphragm has the highest satellite cell contribution to myofiber generation due to the constant activation of this muscle to drive ventilation.^32^ Furthermore, diaphragm satellite cells are molecularly unique from limb muscles in their expression of Pax3.^33^

We also demonstrate that CRISPR/Cas9-mediated genome editing occurs in the satellite cell population *in vivo*, and we quantify the level of gene-editing outcomes, which revealed significantly less gene editing in the satellite cell population compared to bulk muscle (Figures 2 and S1). However, one caveat to this study is the inability to detect edited satellite cells that go on to differentiate into muscle fibers. Therefore, the quantification presented here represents genome-editing levels in the remaining satellite cell population at 8 weeks after treatment and therefore, may underestimate total levels of editing in these cells. Compared to the percentage of satellite cells that underwent Cre-mediated recombination (~60%; Figure 1C), the percentage of total gene editing in satellite cells was significantly lower (up to 1.5%; Figure 2E) despite a comparable dose of AAV, use of the CMV promoter in both studies, same injection route, and same dystrophic muscle context. This may be due to several factors, including the necessity of transduction by two AAV constructs for CRISPR delivery but only one AAV for Cre delivery. Additionally, two separate gRNAs must concurrently induce double-strand breaks for proper exon 23 deletion to occur, and consequently, the intended exon deletion is only a fraction of the total editing events (Figure 2E).^21^ Also, it is probable that Cre-mediated recombination is significantly more efficient than CRISPR-induced gene editing, since Cre acts autonomously, but gene editing requires additional steps of overcoming endogenous DNA repair mechanisms. Accordingly, the ~1.5% total gene-editing frequency does not capture events in which CRISPR induced a double-strand break, and nonhomologous end-joining resulted in perfect repair, which is likely the outcome of most Cas9-induced breaks.

It has been presumed that muscle-specific promoters, which are often constructed from regulatory elements in the creatine kinase promoter, are inactive in satellite cells due to the low expression of creatine kinase in proliferating myoblasts in culture.^9^ By replacing the CMV promoter with various muscle-specific promoters to drive Cre recombinase and delivering equal doses of AAV9, we observed that muscle-specific promoters MHCK7, CK8e, and SpC5-12 were indeed active in satellite cells (Figure 3). Furthermore, these muscle-specific promoters were also able to drive SaCas9 expression to induce gene editing in the satellite cell populations, further supporting their use as an alternative to constitutive promoters, which may create undesired risks, such as off-target genome modification and elicitation of an immune response. Interestingly, the MHCK7 promoter led to the greatest level of dystrophin restoration (Figure 3B) and gene editing (Figure 3C), even relative to CMV. It is not clear at this time what control elements in MHCK7 versus those in CK8e provide a stronger response. The CK7 portion of MHCK7 contains the same highly conserved mouse creatine kinase 5′ enhancer and proximal promoter control elements as those in CK8e, but CK8e lacks several regions of a less highly conserved sequence that were removed to miniaturize the CK8e cassette. MHCK7 also contains a highly conserved, ~190-bp portion of the mouse alpha myosin heavy chain enhancer that contains an MCAT and two GATA control elements that are not present in CK8e.^25^ A potential caveat to the dual reporter mouse is the possibility that differentiating myoblasts may express residual nGFP, while upregulating genes associated with differentiation, such as creatine kinase and myosin heavy chain. However, the high levels of AAV transduction and Cre-mediated recombination with all promoters tested (>10%; Figure 3D) suggest that this small subset of cells does not fully explain the signal we observed.

To further support our conclusions that satellite cells are edited by CRISPR/Cas9 *in vivo*, we used two independent methods: assessing maintenance of dystrophin expression after degeneration (Figure 4) and dystrophin expression following satellite cell transplantation (Figure 5). We demonstrated longevity of dystrophin expression after three rounds of degeneration and regeneration, which may indicate long-term contribution from dystrophin-corrected satellite cells. Importantly, since satellite cells are self-renewing, and there are many satellite cells on each fiber,^20^ even low levels of edited satellite cells may lead to significant levels of dystrophin-positive fibers over time. To directly demonstrate the functionality of edited satellite cells, we performed an engraftment assay, which showed three-fold more dystrophin^+^ fibers in host TA muscles injected with satellite cells isolated from donor TAs treated with AAV9-CMV-CRISPR.

We have previously observed in analysis of AAV8-CMV-CRISPR-treated bulk muscle tissue from *mdx* mice that low levels of editing at the DNA level (~2%) correspond to relatively high levels of exon exclusion at the mRNA level (~59%) and dystrophin-positive muscle fibers (~67%).^5^ We report similar levels here using the Tn5 tagmentation-based quantification. We have speculated that this apparent discrepancy in editing levels and dystrophin expression is explained by protection of the edited dystrophin transcript from nonsense-mediated mRNA decay of the unedited transcript that contains a nonsense mutation in exon 23. Here, we see a similar increase in editing levels in satellite cells when comparing editing at the DNA level (~0.05% deletions; Figure 2E) to edited RNA transcripts (~0.5%–1%; Figure 2F). This observation is also consistent with recent reports that surprisingly observe dystrophin expression in satellite cells.^27^

In summary, this study builds upon previously reported evidence of satellite cell transduction and CRISPR/Cas9-induced gene editing and provides new insights into choice of AAV serotype and promoter. With the use of an unbiased Tn5 tagmentation-based sequencing method, we provide quantifications of the amount of gene editing in satellite cells. There is significant interest in developing enhanced delivery vehicles for CRISPR to muscle, including evolved or engineered AAV capsids^34^ and nonviral nanoparticle strategies that overcome challenges of viral vectors.^35^^,^^36^ Delivery to satellite cells by these methods may be an important consideration for their ultimate success in the context of inherited muscular dystrophies. Further optimization of gene-editing technologies and delivery methods should focus on enhancing satellite cell gene editing to provide a long-term therapeutic effect for DMD patients.

## Materials and Methods

### Plasmid Design and AAV Production

CMV-driven Cre recombinase-containing AAV constructs were purchased from the Penn Vector Core. The CMV-Cre plasmid was also purchased from the Penn Vector Core and used to generate CK8e-Cre, SPc5-12-Cre, and MHCK7-Cre AAV transfer plasmids. For CRISPR experiments, an AAV transfer plasmid containing CMV-SaCas9-3×HA-bGHpA was acquired from Addgene (plasmid #61592). CMV was removed, and muscle-specific promoters were cloned into this plasmid to generate CK8e-, SPc5-12-, and MHCK7-driven SaCas9 transfer plasmids. AAV transfer plasmid containing two gRNA expression cassettes for mouse exon 23 excision driven by the human U6 promoters was used to prepare recombinant AAV, as previously described.^5^ Intact ITRs were confirmed by SmaI digestion before AAV production on all vectors. Multiple batches of AAV were produced and titers measured by qRT-PCR with a plasmid standard curve to ensure equal dosage within studies.

### Animals

The mouse strains C57BL/10ScSn-*Dmd^mdx^*/J (*mdx*) and B6.Cg-*Gt(ROSA)26Sor^tm9(CAG-tdTomato)Hze^*/J (*Ai9)* were obtained from The Jackson Laboratory. Pax7nGFP mice were generated by knocking in a nGFP signal into the first exon of the endogenous Pax7 and were kindly provided by S. Tajbakhsh (Institut Pasteur). Non-obese diabetic (NOD). severe combined immunodeficiency (SCID).gamma mice were obtained from the Duke Cancer Center Isolation Facility (CCIF) Breeding Core. Pax7nGFP(+/−);Ai9(+/−);mdx(+/0) males were used for the Cre studies. All experiments involving animals were conducted with strict adherence to the guidelines for the care and use of laboratory animals of the National Institutes of Health (NIH). All experiments were approved by the Institutional Animal Care and Use Committee (IACUC) at Duke University.

### *In Vivo* AAV Administration

All mice used for these studies were males injected at 6–8 weeks of age. For comparison of AAV1, -2, -5, -6.2, -8, and -9 in Cre-mediated recombination of satellite cells, Pax7nGFP;Ai9;mdx mice were administered locally into the TA muscle with 40 μL of 4.72E+11 vg or systemically via tail-vein injection with 200 μL of 2E+12 vg. At 8 weeks postinjection, mice were euthanized, and muscle was collected for analysis by flow cytometry and immunofluorescence staining.

For comparison of Cre-mediated recombination of satellite cells between constitutive and muscle-specific promoters, Pax7nGFP;Ai9;mdx was administered locally into the TA muscle with 40 μL of 4.00E+10 vg of AAV9 CMV-, CK8e-, SPc5-12-, or MHCK7-driven Cre.

For AAV9-CRISPR experiments, mice were injected locally with 7E+11–1E+12 vg per vector or systemically with 5E+12 vg per vector.

For serial injury experiments, *mdx* mice were injected with 1E+12 vg per vector of AAV9-CMV-CRISPR constructs into the TA muscle. 4 weeks after injection, the TA was subjected to injury with 50 μL of BaCl_2_. The muscle was allowed to recover for 2 weeks before subsequent additional BaCl_2_ injuries. Muscle was harvested 2 weeks after the last BaCl_2_ injury.

### AAV-CRISPR Cell Transplantation Experiments

For engraftment experiments, Pax7nGFP;mdx mice were injected with a total of 2E+12 vg per CRISPR vector into the hindlimb. (TA, gastrocnemius, and quadricep muscles were injected.) Control Pax7nGFP;mdx mice were injected with PBS. 8 weeks later, the injected hindlimb was collected, and satellite cells were isolated via enzymatic digestion and sorting. 20,000–40,000 satellite cells were isolated per mouse, and cells were spun down and resuspended in 15 μL Hank’s balanced salt solution supplemented with 10 ng/mL of basic fibroblast growth factor (bFGF).

2 days prior to intramuscular cell transplantation, recipient *mdx* mice were anesthetized with isoflurane, and one hindlimb received an 18-Gy dose of irradiation using an X-RAD 320 biological irradiator. 1 day prior to transplantation, mice began an immunosuppression regimen with daily i.p. injections of tacrolimus (Prograf, 5 mg/kg). Satellite cells sorted from Pax7nGFP mice, treated with AAV9-CMV-CRISPR or PBS 8 weeks prior, were injected into the TA muscle of recipient *mdx* mice. 4 weeks after transplantation, mice were euthanized, the TA muscles were harvested for genomic DNA extraction, and a portion of tissue was embedded for sectioning and staining for dystrophin expression.

### Satellite Cell Isolation

For local intramuscular studies, the muscle was harvested and cut into small pieces. Muscle was enzymatically digested with 0.2% collagenase II (Invitrogen; 17101-015) in DMEM (Invitrogen) for 1 h, followed by a 30-min digest with 0.2% Dispase (Invitrogen; 17105-041). Cells were strained through a 30-μm filter and sorted by GFP expression on a SONY SH800 flow cytometer. Cells were collected by centrifugation, and genomic DNA was isolated immediately by phenol-chloroform extraction.

### Genomic DNA Analysis

Genomic DNA from mouse muscle was extracted with the DNeasy kit (QIAGEN). Exon 23 deletion was assessed, as previously described.^5^ Tn5-mediated target enrichment and sequencing were performed, as previously described,^21^ using the Nextera DNA Flex Library Prep Kit (Illumina).

### Histology and Immunofluorescence

Harvested muscles were mounted and frozen in optimal cutting temperature (OCT) compound cooled in liquid nitrogen. Serial 10 μm cryosections were collected. Cryosections were fixed with 2% paraformaldehyde (PFA) for 5 min and permeabilized with PBS + 0.2% Triton-X for 10 min. Blocking buffer (PBS supplemented with 5% goat serum, 2% BSA, Mouse on Mouse [M.O.M.] blocking reagent, and 0.1% Triton X-100) was applied for 1 h at room temperature. Samples were incubated overnight at 4°C with a combination of the following antibodies: Pax7 (1:5; Developmental Studies Hybridoma Bank [DSHB]), MANDYS8 (1:200; D8168; Sigma), laminin (1:200; L9393; Sigma), red fluorescent protein ([RFP] 1:1,000; 600-401-379; Rockland Immunochemicals). Samples were washed with PBS for 15 min and incubated with compatible secondary antibodies diluted 1:500 from Invitrogen and 4′,6-diamidino-2-phenylindole (DAPI) for 1 h at room temperature. Samples were washed for 15 min with PBS, and slides were mounted with ProLong Gold Antifade Reagent (Invitrogen) and imaged using conventional fluorescence microscopy. Images (60×) were taken with a confocal microscope.

### Western Blots

Protein analysis and western blot muscle biopsies were disrupted with a BioMasher (Takara) in radioimmunoprecipitation assay (RIPA) buffer (Sigma) with a proteinase inhibitor cocktail (Roche) and incubated for 30 min on ice with intermittent vortexing. Samples were centrifuged at 16,000 × *g* for 30 min at 4°C, and the supernatant was isolated and quantified with a bicinchoninic acid assay (Pierce). Protein isolate was mixed with in NuPAGE loading buffer (Invitrogen) and 10% β-mercaptoethanol and boiled at 100°C for 10 min. Samples were flash frozen in liquid nitrogen for future analysis. 25 μg total protein per lane was loaded into 4%–12% NuPAGE bis-Tris gels (Invitrogen) with MOPS buffer (Invitrogen) and electrophoresed for 45 min at 200 V. Protein was transferred to nitrocellulose membranes for 1 h in 1× Tris-glycine transfer buffer containing 10% methanol and 0.01% SDS at 4°C at 400 mA. The blot was blocked in 5% milk-Tris-buffered saline Tween 20 (TBST) and probed with anti-HA (1:1,000; 901502; BioLegend) or glyceraldehyde 3-phosphate dehydrogenase ([GAPDH] 1:5,000; 2118S; Cell Signaling Technology) overnight in 5% milk-TBST at 4°C. Blots were then incubated with mouse or rabbit horseradish peroxidase-conjugated secondary antibodies (Santa Cruz) for 1 h in 5% milk-TBST. Blots were visualized using WesternC enhanced chemiluminescence (ECL) substrate (Bio-Rad) on a ChemiDoc chemiluminescent system (Bio-Rad).

## Author Contributions

J.B.K. and C.A.G. designed experiments. J.B.K., A.R.E., A.V., and J.D.B. performed the experiments. G.D. and A.A. generated and provided the AAV used for the experiments. S.D.H. provided muscle-specific promoters. J.B.K. analyzed the data with help from all authors. J.B.K. and C.A.G. wrote the manuscript with contributions by all other authors.

## Conflicts of Interest

J.B.K. and C.A.G. have filed patent applications related to technologies for genome engineering and cell reprogramming. C.A.G. is a scientific advisor to Sarepta Therapeutics, Tune Therapeutics, Levo Therapeutics, and Iveric Bio and a cofounder of Element Genomics and Locus Biosciences. C.A.G. and J.B.K. are cofounders of Tune Therapeutics. A.A. is a cofounder and advisor to Stride Bio and Torque Bio.

## Supplemental Information

Document S1. Table S1 and Figure S1

Document S2. Article plus Supplemental Information
