# Supplementary material for: In Vivo Gene Editing of Muscle Stem Cells with Adeno-Associated Viral Vectors in a Mouse Model of Duchenne Muscular Dystrophy
Source: Mol Ther Methods Clin Dev. 2020 Sep 28;19:320–9. doi: 10.1016/j.omtm.2020.09.016 (PMC7581966; doi:10.1016/j.omtm.2020.09.016)
Supplement: Document S1. Table S1 and Figure S1 [file mmc1.pdf]

OMTM, Volume *19*

Supplemental Information

In Vivo Gene Editing of Muscle Stem Cells with Adeno-Associated Viral Vectors in a Mouse Model of Duchenne Muscular Dystrophy

Jennifer B. Kwon, Adarsh R. Ettyreddy, Ashish Vankara, Joel D. Bohning, Garth Devlin, Stephen D. Hauschka, Aravind Asokan, and Charles A. Gersbach

Table S1. Oligonucleotide sequences

Primer Name	Primer sequence (5'-3')
**Deletion PCR Primers**	
DMDin22 -F	TTTGTTGATTCTAAAAATCCCATGT
DMDin23 -R	GGACTGAAGAACTTGGAGAAGGA
NestDMDin22-F	GTTTCACTGTAGGTAAGTTAAAATG
NestDMDin23-R	GGAGCAACTTTGGAAAGTAAA
**Nextera Sequencing Primers**	
DMDin22 Tn5-F	AAGCAGTGGTATCAACGCAGAGTAC ATTGCTTTATCAGATATTCTACT
DMDin23 Tn5-F	AAGCAGTGGTATCAACGCAGAGTAC TTCAAAAAGGAAGATGGTACAGTGTT
DMDEx22 Tn5-F	AAGCAGTGGTATCAACGCAGAGTAC GGATCCAGCAGTCAGAAAGC
DMDEx24 Tn5-F	AAGCAGTGGTATCAACGCAGAGTAC TCACCAACTAAAAGTCTGCATTG
Nextera-R	GTCTCGTGGGCTCGGAGATGTGTATAAGAGACAG
i5+custom adapter forward primer	AATGATACGGCGACCACCGAGATCTACACGCCTGTCCGCGGAAGCAGTGGTATCAACGCAGAGTAC
Barcoded reverse primer	CAAGCAGAAGACGGCATACGAGAT [Barcode] GTCTCGTGGGCTCGG
Custom sequencing primer	CGGAAGCAGTGGTATCAACGCAGAGTAC

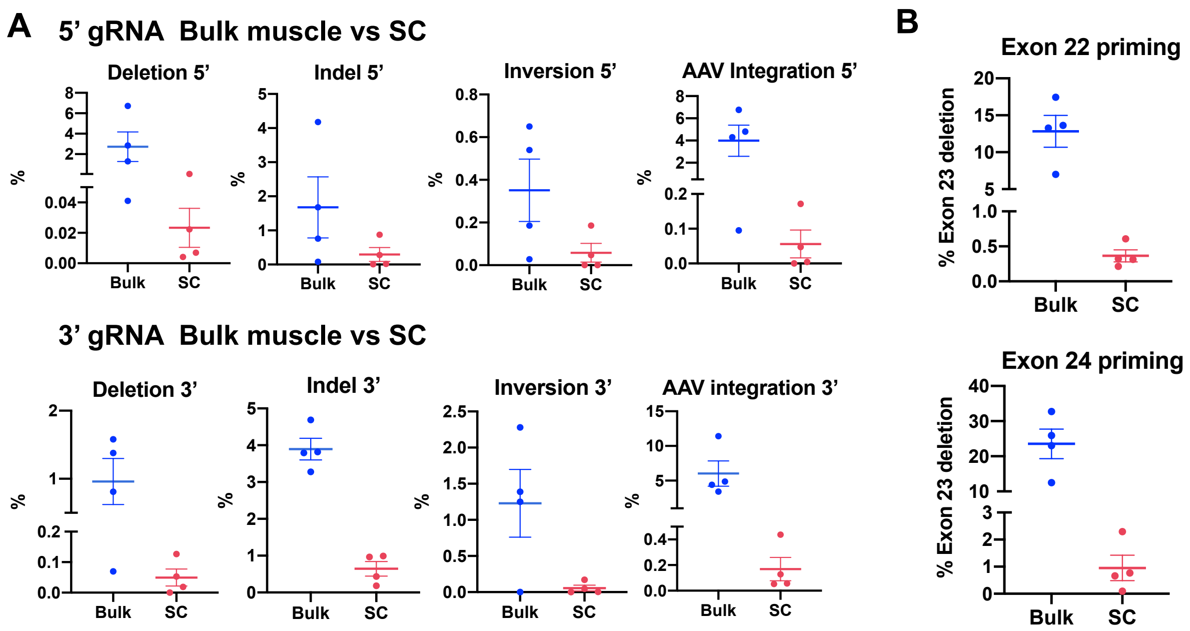

Figure S1. (**A**) Unbiased sequencing of satellite cell or bulk muscle genomic DNA after AAV9-CMV-CRISPR local administration quantifies the level of editing events for various gene-editing outcomes (**B**) Unbiased sequencing of satellite cell or bulk muscle mRNA after AAV9-CMV-CRISPR local administration quantifies the level of exon 23 deletion. (mean ± SEM, n = 4 mice).
